# Pure crystal orientation and anisotropic charge transport in large-area hybrid perovskite films

**DOI:** 10.1038/ncomms13407

**Published:** 2016-11-10

**Authors:** Namchul Cho, Feng Li, Bekir Turedi, Lutfan Sinatra, Smritakshi P. Sarmah, Manas R. Parida, Makhsud I. Saidaminov, Banavoth Murali, Victor M. Burlakov, Alain Goriely, Omar F. Mohammed, Tom Wu, Osman M. Bakr

**Affiliations:** 1 Division of Physical Sciences and Engineering (PSE), KAUST Solar Center, Materials Science and Engineering, King Abdullah University of Science and Technology (KAUST), Thuwal 23955-6900, Saudi Arabia; 2 Materials Science and Engineering, King Abdullah University of Science and Technology (KAUST), Thuwal 23955-6900, Saudi Arabia; 3 Mathematical Institute, University of Oxford, Woodstock Road, Oxford OX2 6GG, UK

## Abstract

Controlling crystal orientations and macroscopic morphology is vital to develop the electronic properties of hybrid perovskites. Here we show that a large-area, orientationally pure crystalline (OPC) methylammonium lead iodide (MAPbI_3_) hybrid perovskite film can be fabricated using a thermal-gradient-assisted directional crystallization method that relies on the sharp liquid-to-solid transition of MAPbI_3_ from ionic liquid solution. We find that the OPC films spontaneously form periodic microarrays that are distinguishable from general polycrystalline perovskite materials in terms of their crystal orientation, film morphology and electronic properties. X-ray diffraction patterns reveal that the film is strongly oriented in the (112) and (200) planes parallel to the substrate. This film is structurally confined by directional crystal growth, inducing intense anisotropy in charge transport. In addition, the low trap-state density (7.9 × 10^13^ cm^−3^) leads to strong amplified stimulated emission. This ability to control crystal orientation and morphology could be widely adopted in optoelectronic devices.

## Abstract (web summary)

Lead halide perovskites are developed for a number of optoelectronic applications. Here, Cho *et al*. use a thermal gradient to achieve directional crystallization of methyl ammonium lead iodide films. The periodic microarrays exhibit anisotropic charge transport properties.

## Graphical abstract

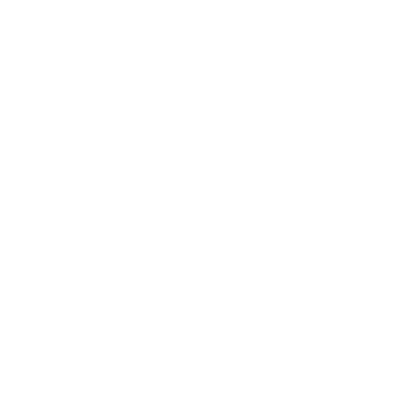

Organolead halide (APbX_3_) perovskites have characteristics that make them attractive for inclusion in several optoelectronic devices, such as solar cells^1^,^2^,^3^,^4^,^5^,^6^, photodetectors^7^,^8^,^9^,^10^,^11^,^12^ and light-emitting diodes^13^,^14^, as well as lasing applications^15^. The performances of these perovskite-based optoelectronic devices is determined mainly by their electrical and optical properties and the film processing conditions; for example, film morphology and crystal orientation are central to determining the fundamental properties of organolead halide perovskite materials^16^,^17^,^18^,^19^,^20^. Most investigations with respect to the film formation of perovskites have focused on optimizing grain size and crystallinity during the deposition of perovskite films, which is critical for reducing grain boundaries and defect density, thus suppressing charge recombination and increasing diffusion lengths of the charge carriers. By virtue of these studies on materials and device engineering, the power conversion efficiencies (PCEs) of polycrystalline perovskite solar cells have surpassed 20% (ref. 21). Despite these triumphs in perovskite photovoltaic devices, a significant gap exists in understanding the charge transport properties of perovskite materials, especially their relationship with micro or nano-structure of the materials.

Recent reports of long charge carrier diffusion lengths and low trap-state densities from methylammonium lead halide (MAPbX_3_) based single crystal perovskites, which could lead to a new generation of highly efficient optoelectronic devices^22^,^23^, have been a key motivator for researchers to bridge the performance and properties gap between perovskite single crystals and their polycrystalline film counterparts. However, the fabrication of fully covered large area devices, which is readily available for solution-processed polycrystalline perovskites, is extremely challenging for single crystals and has so far not been achieved. In the case of solution processed polycrystalline perovskites, precise control of crystal orientations and macroscopic morphology needs to be addressed to narrow down the wide disparity in materials properties between single and polycrystalline perovskites.

Here we demonstrate that introducing a thermal gradient on the growth-substrate controls the macroscopic solidification direction and provides a powerful strategy for confining crystal orientations, as well as rationally tuning charge transport properties in organolead halide perovskites. We used an ionic liquid^24^ as a solvent because it affords the sharp liquid-to-crystalline solid transition of MAPbI_3_ from the ionic liquid solution, as opposed to traditional perovskite processing solvents (for example, DMSO or DMF) generating plumbate intermediates during crystallization^25^. The resulting orientationally pure crystalline (OPC) films, which are comprised of periodic microarrays with strong orientational preference to the (112) and (200) planes, are useful for a wide range of optoelectronic applications, such as highly efficient phototransistors and optical gain media. We explored the role played by the macroscopic alignment in influencing charge transport properties. Remarkably, transistor devices constructed of OPCs show giant anisotropy (three orders of magnitude difference) in their field effect mobility depending on the orientation of perovskite films.

## Results

### Preparation of orientationally pure crystalline perovskite films

We implemented an ionic liquid (methylammonium formate, MAFa) as a solvent in which both methylammonium iodide (MAI) and PbI_2_ are completely soluble and transform into perovskites with no intermediate structures^24^. The chemical structure of MAFa is similar to that of MAI and it can form hydrogen bonds with both MAI and PbI_2_ (ref. 24). Thus we expect a high crystal quality to be maintained in the absence of the formation of plumbate intermediates via reversible inclusion of solvent molecules, such as DMSO and DMF (refs 24, 25). We exploited these advantages to design a temperature-gradient-assisted solidification process to grow large-area, high-quality MAPbI_3_ OPC films.

**Figure 1:**
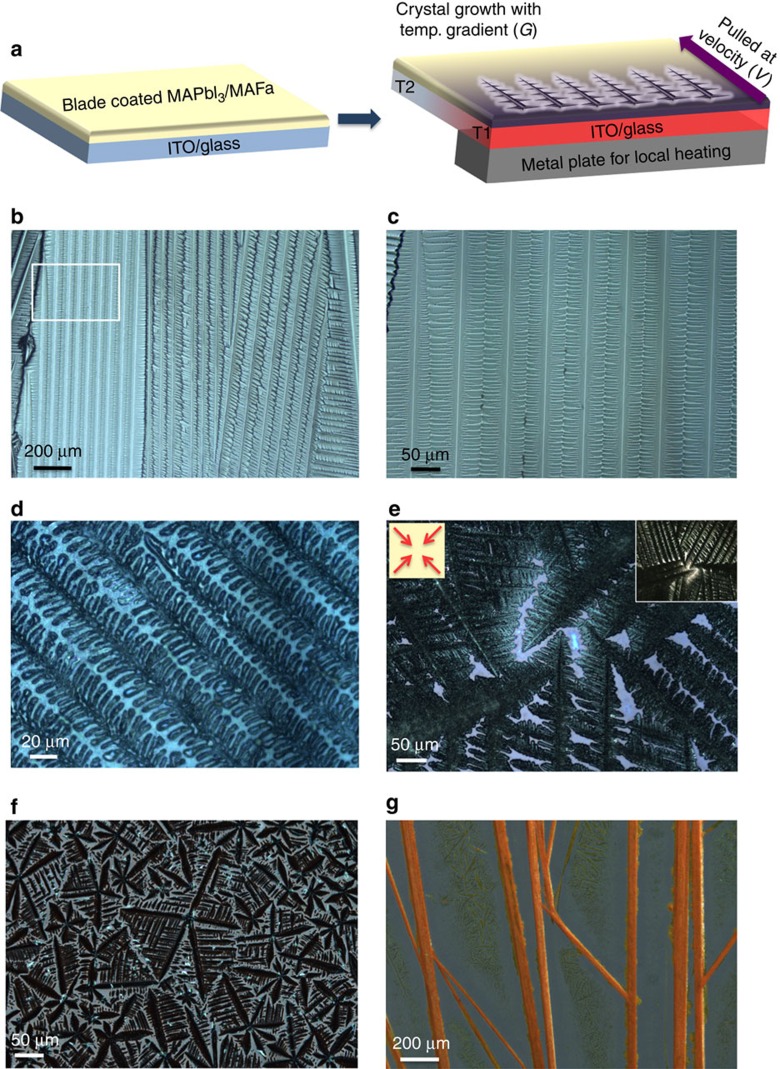
Growth of orientationally pure crystalline (OPC) films. (**a**) Schematic representation of the thermal-gradient-assisted directional crystallization process of a MAPbI_3_ perovskite. A solution is pulled at a constant velocity, *V*, through a thermal gradient, *G*, generated by local heating at T1. (**b**) Optical microscope image of the aligned OPC films of the perovskite and (**c**) the magnified image of the highlighted area in (**b**) (white square). (**d**) The intermediate stage of microarray growth in liquid films showing the secondary branches growing from the propagating main backbone. (**e**) Isotropically growing microarrays by homogeneous annealing of the substrate. Insets show the same sample at a lower magnification (upper right) and the direction of its growth (upper left). (**f**) Spherulitic dendrites generated by lowering concentration with small thermal gradient or homogeneous annealing. (**g**) Optical microscope image of directionally grown MAPbBr_3_ wires using the thermal gradient method.

Figure 1a illustrates our thermal gradient-assisted directional crystallization process. First, the liquid film was deposited by the blade-coating method from the MAI:PbI_2_ (1:1 molar ratio) solution in MAFa (25 wt%) onto an indium tin oxide (ITO)-coated glass substrate. The liquid film was pulled at a constant velocity, *V*, through a thermal gradient, *G*, which was generated by local heating. The resultant structure has two distinct observable growth patterns: stripes (main backbones) that propagate and form along the thermal gradient, presumably due to Mullins–Sekerka instability^26^, and branches that grow perpendicular to the thermal gradient. To grow well-aligned OPC films forming periodic microarrays, the thermal gradient at the solid–liquid interface needs to be carefully controlled, assuring sequential solidification along the axial direction. We observed that certain conditions are necessary for directional growth of the microarrays and that variations in concentration, temperature and pulling velocity produce irregular or non-directional dendritic patterns. By balancing both temperature and concentration, we were able to grow well-aligned perovskite OPC films on a planar substrate over several cm^2^ (Supplementary Fig. 1). Figure 1b shows the optical microscope image of the aligned OPC films and the highlighted area is enlarged in Fig. 1c. We found optimum solidification conditions at *V*=2.1 μm s^−1^ and *G*=0.67 °C mm^−1^. Figure 1d shows the intermediate state of arrays growing in liquid films. Similar to solutions in DMSO and DMF, in which flower-like structures have been previously been reported to grow^27^, we observed the formation of spherulitic dendrites with blade-coated perovskite films in MAFa when the propagating solid–liquid interface was perturbed by any air or thermal turbulence (caused by moving the substrate or by air flow over the substrate) during directional solidification. Homogeneous annealing of the substrate produces isotropically grown microarrays (Fig. 1e), and further lowering of the concentration ∼7 wt% with a small temperature gradient *G*, or homogeneous annealing leads to spherulitic dendrites (Fig. 1f). We were not able to generate aligned films at high concentrations greater than 50 wt%. See Supplementary Fig. 2 for details. In the case of MAPbBr_3_, 1–2 cm long micro-wires were generated by the thermal gradient method (Fig. 1g) compared with the randomly oriented microwires generated by homogeneous annealing (Supplementary Fig. 3). Although we were unable to make fully covered films from MAPbBr_3_ solution in MAFa (Supplementary Fig. 3), the long perovskite wires we were able to generate had characteristics of directional growth unlike the previously reported randomly oriented perovskite nanowires^28^,^29^.

### Structural characterization

**Figure 2:**
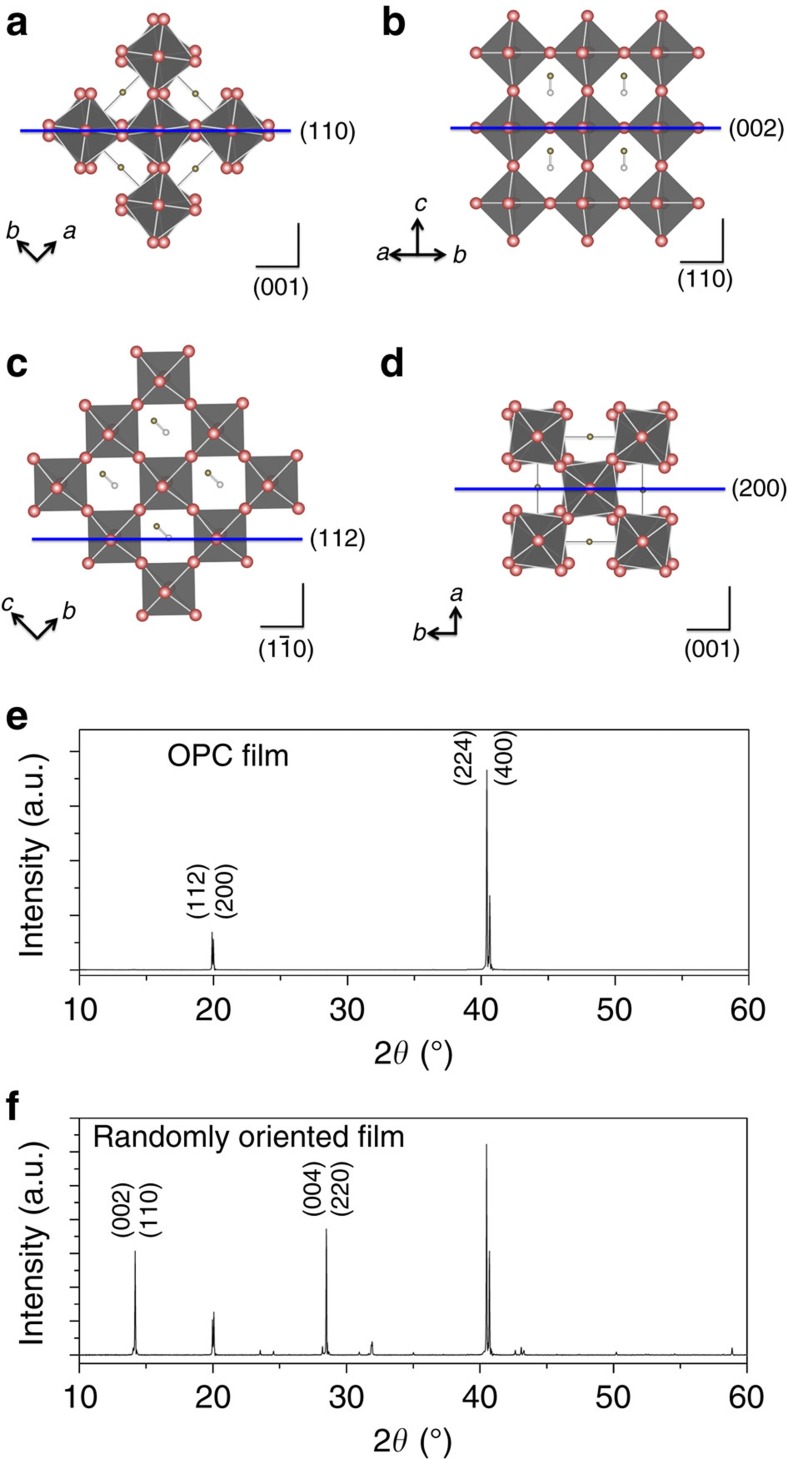
X-ray diffraction analysis. Unit cell of the tetragonal MAPbI_3_ perovskite showing (**a**) (110), (**b**) (002), (**c**) (112), (**d**) (200) planes indicated by blue lines. (**e**) X-ray diffraction of the OPC and (**f**) randomly oriented perovskite films. The aligned film only shows the (112) (200) and (224) (400) planes, whereas the randomly oriented film shows general X-ray diffraction patterns.

We demonstrate that thermal-gradient-assisted directional crystallization is a compelling strategy for confining crystal orientations. Figure 2a–d shows the crystal structure of the tetragonal MAPbI_3_ perovskite with (110), (002), (112) and (200) planes, respectively. Fig. 2e and f show thin film X-ray diffraction patterns of OPC and randomly oriented spherulitic MAPbI_3_ films, respectively; both show the tetragonal structure of the perovskites. We observed that the X-ray diffraction patterns of the aligned OPC perovskite films show strong peaks at 2*θ*=19.90° and 20.00° corresponding to (112) and (200) planes, respectively, and peaks at 2*θ*=40.42° and 40.64° corresponding to (224) and (400) planes, respectively. This indicates that MAPbI_3_ crystals in OPC films orient mainly along the (112) or (200) directions parallel to the substrate. Clearly, there is a preferential orientation of the crystals such that edges of the PbI_6_ octahedron are in contact with the substrate. From detailed X-ray diffraction analysis, we found peaks with nearly negligible intensities, with respect to the baseline signal, in the (110) and (002) planes, as well as other tiny peaks for different planes (Supplementary Fig. 5). Interestingly, such strictly confined crystal orientations have been reported mainly from epitaxially grown films^30^ and single crystal perovskites^31^. Recent reports have elucidated the transformation pathways and intermediates composed of perovskite precursors and solvent molecules. The structure of these intermediates alters the nucleation dynamics and crystal formation. Recently, Miyadera *et al*. found that the crystal orientation was confined exclusively in two specific directions according to the orientation of the initial PbI_6_ octahedral framework on the substrate in the early stage (up to 20 s) of the crystallization process^32^. This suggests that a pure crystal orientation could be obtained via careful control of the nucleation of perovskite from PbI_2_ and the efficient intercalation of MAI molecules into the growing PbI_2_ framework. It is clear that the MAFa is a good solvent to dissolve PbI_2_ and more so for MAI^24^. Due to the ionic character of MAFa and its strong interaction (solvation) between COO^−^ and Pb^2+^ and CH_3_NH_3_^+^ and I^−^, it dissolves PbI_2_ better than other common organic solvents such as DMF and DMSO. This might be partially responsible for the slow crystallization of lead halide during our directional crystallization process because of the low degree of supersaturated Pb^2+^ and X^−^. In addition, an excellent solubility of MAI in MAFa could be responsible for the efficient intercalation process of MA cations into the lead halide crystal frameworks, resulting in the formation of compact and homogeneous crystal nuclei. The initial PbI_6_ octahedral crystals could be rearranged in our ionic liquid systems by Ostwald ripening^33^ during thermal annealing in order to reduce the surface energy and form thermodynamically more stable perovskite structures. The unique (112) and (200) crystal orientations in our system are probably intrinsic to this new class of materials combination and further controlled when their crystallization process is precisely manipulated. Further investigation is required to fully ascertain the exact reasons for MAFa's generation of OPC films of specific directions.

In contrast to the OPC films, randomly oriented sperulitic films generally show X-ray diffraction patterns with a couple of major peaks corresponding to the (110), (002), (112) and (220) planes (Fig. 2f). Note that our films, which were prepared in ambient conditions, do not exhibit any observable peak at 2*θ*=12.65°, corresponding to the (001) diffraction peak of PbI_2_, revealing the complete conversion of a perovskite film from precursors. In the case of aligned MAPbBr_3_ macro-wires, they exhibit typical X-ray diffraction patterns similar to those observed from polycrystalline films (Supplementary Fig. 4). The different solubility of PbBr_2_ in MAFa and fast nucleation/growth rate, which can interrupt the desired reaction rate required for a slow and balanced crystallization process, could be responsible for the different growth habit of MAPbBr_3_ crystals (as compared with MAPbI_3_).

**Figure 3:**
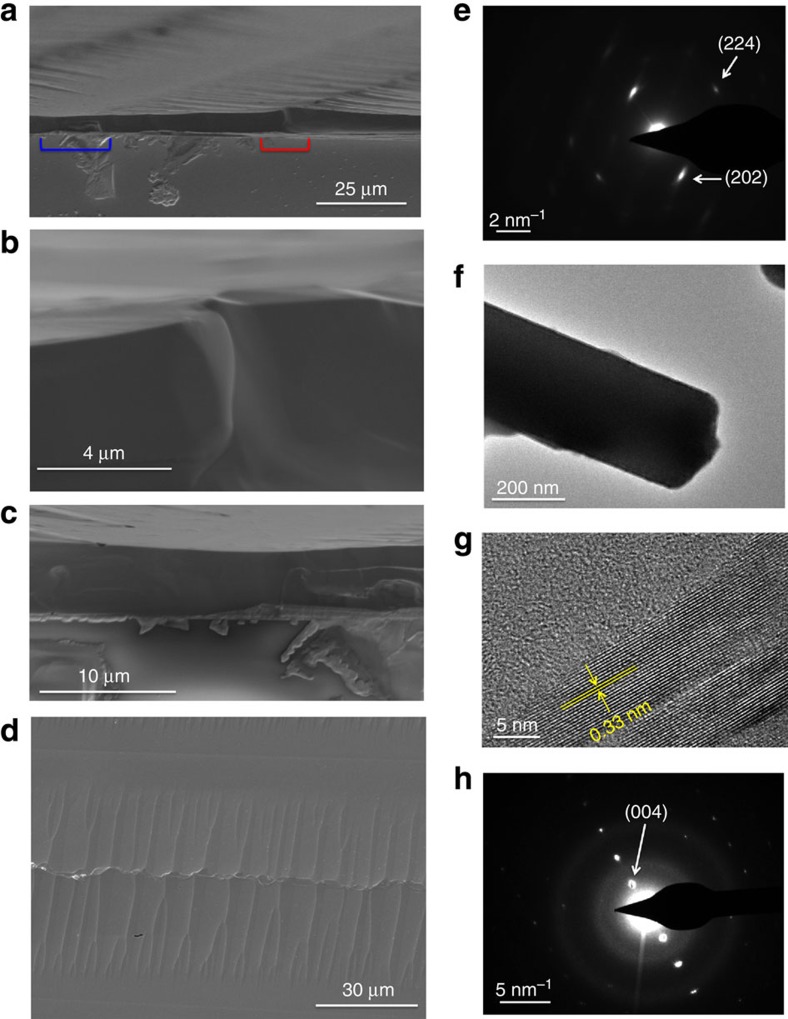
SEM and TEM analysis. (**a**) Cross-sectional SEM image of the aligned OPC film. (**b**) Cross-sectional area where two branches meet and form grain boundaries (the area in red line in **a**). (**c**) Cross-sectional area with no grain boundaries observed in the main backbone area (the area in blue line in **a**). (**d**) Planar SEM image of the aligned film. (**e**) The electron diffraction corresponding to the entire TEM image of a thick specimen as shown in **f**. (**f**) The TEM image of a thick specimen. (**g**) The TEM image of a thin specimen and (**h**) its electron diffraction image. Inter-planer spacing of 0.33 nm can be indexed to the (004) plane.

Figure 3a shows a cross-sectional scanning electron microscopy (SEM) image of the aligned OPC film. Figure 3b (the area in red line in Fig. 3a) shows the cross-sectional area where two branches meet and form grain boundaries, while the cross-sectional image in Fig. 3c (the area in blue line in Fig. 3a ) shows that no grain boundaries are present in the main backbone area. We believe that the structural defect where the side branches join could be responsible for the anisotropic charge transport properties. In other words, those grain boundaries impede charge transport more severely when we measure the mobilities of carriers in a direction normal to the main backbone. We will discuss this in detail in film orientation dependent charge transport section. Meanwhile, planar SEM images illustrate aligned OPC films with clean top surfaces (Fig. 3d). The structural properties of these films were further investigated by transmission electron microscopy (TEM) and selected area electron diffraction analysis. To prepare TEM samples, the OPC film dispersions in toluene were sonicated for 5 s and then deposited onto a copper grid. Figure 3e shows the electron diffraction pattern observed from a thick specimen of OPC microarrays on the copper grid. It shows clear spots that can be indexed to the (224) and (202) planes of the tetragonal MAPbI_3_ phase. This diffraction pattern corresponds to the entire area of the image as shown in Fig. 3f. Figure 3g,h show the TEM image of a thin specimen and its electron diffraction image, respectively. The crystalline structure with a lattice spacing of 0.33 nm was observed. This lattice fringe could be indexed to the (004) plane of the tetragonal MAPbI_3_. A small branch separated from the microarrays shows the same lattice spacing (Supplementary Fig. 7).

### Optical properties

**Figure 4:**
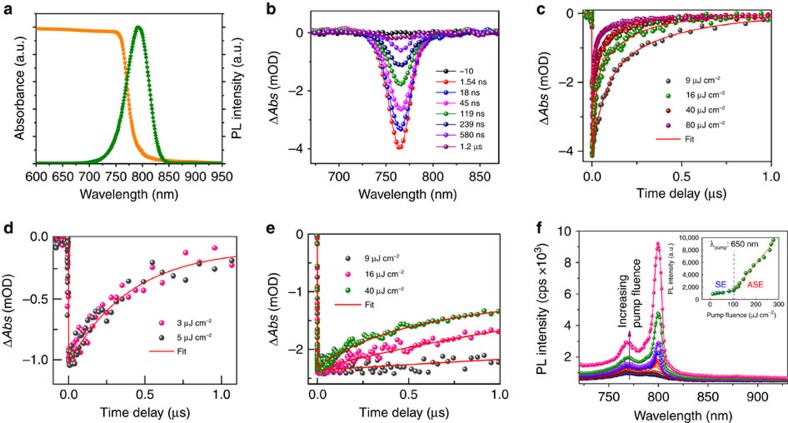
Optical properties. (**a**) Steady-state absorption and PL spectra of the OPC film. The absorption band edge and the PL maximum are located at 780 nm (1.59 eV) and 795 nm, respectively. (**b**) TA spectra of the aligned OPC film recorded following 650 nm excitation at various pump-probe delay times. (**c**) ns-TA decay profiles of 765 nm GSB recovery at various pump intensities. Higher excitation intensities resulted in an increased rate of the recombination. Traces are normalized to the maximum bleach signal at each excitation energy density. The data were fitted with a bi-exponential decay function (see Methods). (**d**) Kinetic profiles of 765 nm GSB recovery at low pump intensities. The data were fitted with a single-exponential decay function. (**e**) fs-TA decay profiles of 765 nm GSB recovery with various pump intensities. (**f**) Steady-state PL spectra from the aligned OPC film photoexcited using 650 nm (35 fs and 1 kHz pump pulses) light with increasing pump fluence approximately from 15 to 270 μJ cm^−2^. Transition from SE to ASE was observed at 102 μJ cm^−2^ (inset figure).

We investigated the steady-state optical properties of the aligned OPC films (Fig. 4a) in order to find the absorption band edge and the photoluminescence (PL), which were observed at 780 nm (1.59 eV) and 795 nm, respectively. See Supplementary Fig. 6 for Tauc plot from the absorption spectrum. Next, we investigated the relaxation processes in the OPC films by means of transient absorption (TA) spectroscopy. The slow recovery of ground state bleaching we observed represents the decay of photogenerated free charge carriers. On optical excitation at 650 nm, the aligned films exhibit ground-state bleach at 765 nm, which is attributed to the band-edge transitions of the perovskite (Fig. 4b). Fig. 4d shows the normalized kinetic traces of the OPC films at various low pump fluences of <6 μJ cm^−2^. The fitting of the time profiles from ns-TA data give a single exponential decay with a characteristic time constant of 430±20 ns. In other words, at very low fluences of the absorbed photons monomolecular carrier recombination (trap-state mediated recombination) is observed^34^,^35^,^36^. At high pump fluences, the fitting of the decay profiles of OPC films shows two lifetime components (Fig. 4c and Supplementary Table 1). The fast initial decay component increases significantly with increasing the pump fluence, which is attributed to higher order recombination processes^37^. Such a fast recombination is also evident from the femtosecond transient absorption experiments, as shown in Fig. 4e.

We reveal that our aligned OPC film is a good optical gain medium exhibiting strong coherent light-emission properties owing to its low trap-state density and excellent crystallinity. Amplified stimulated emission (ASE) measurements were carried out on the OPC films after pumping with 35 fs laser pulses with tunable wavelengths in a cavity-free configuration. Figure 4f shows how the emission spectra behave with increasing pump fluence at 650 nm laser excitation pulses. At low pump fluence, we observed a broad emission spectrum corresponding to spontaneous emission (SE). As pump fluence was gradually increased, SE was accompanied by a sharp peak (transition to ASE) on the lower energy side of the broad bands, centered at 798 nm. A slightly red-shifted ASE peak with respect to the PL peak has been correlated with balanced optical gain and absorption^15^. A sharp transition from SE to ASE was found at a pump fluence of 102 μJ cm^−2^ (inset in Fig. 4f), which corresponds to the ASE threshold.

**Figure 5:**
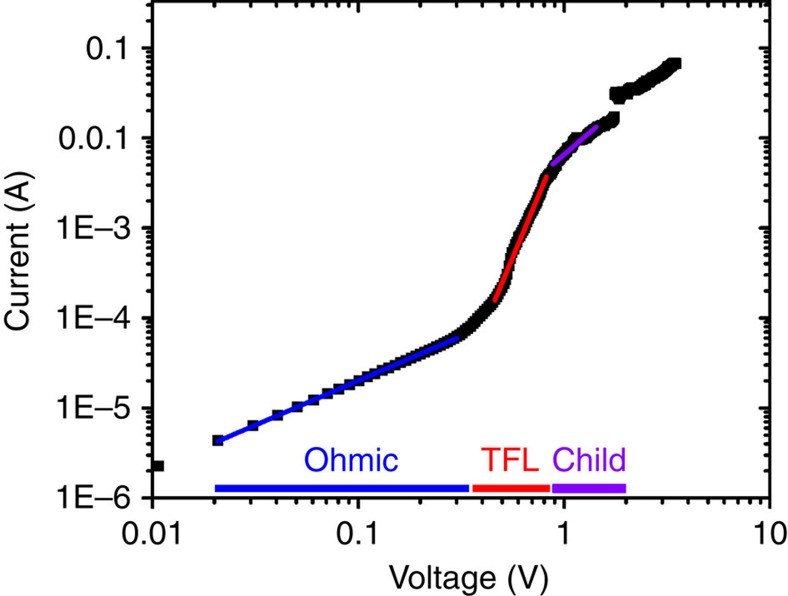
Electrical Properties. Current–voltage characteristics of the OPC film showing Ohmic, TFL and Child region. The trap density was estimated from the TFL region. The onset voltage of the TFL region is ∼0.34 V.

In general, low defect densities and slow multi-particle non-radiative recombination rates are critical factors for achieving low ASE thresholds^38^. Although bimolecular recombination is very low in highly crystalline MAPbI_3_ perovskite films^36^, it may not be negligible in the trap-filled limit regime under high excitation fluence or high external electric field. The trap-state density of the OPC film was extracted from the current–voltage data: *N*_t_=2*ɛ*_0_*ɛ*_r_*V*_TFL_/*qL*^2^, where *ɛ*_0_ and *ɛ*_r_ are the absolute and relative dielectric constants, respectively, *V*_TFL_ is the onset voltage of the trap-filled limit region, *q* is the elemental charge, and *L* is the thickness of the film. We measured *ɛ*_r_ of the OPC perovskite films by the capacitance-voltage measurement with an ITO/perovskite/Au structure. The dielectric constant, averaged in the frequency range from 100 kHz to 1 MHz, (plateau region) is 33.7±0.2 (See Supplementary Fig. 12 and Methods section for more details), which is comparable with previously reported values^39^,^40^,^41^,^42^,^43^. Fig. 5 shows the current–voltage characteristics of the perovskite OPC film with an ITO/perovskite (4 μm)/Au (100 nm) structure. The onset voltage of the trap-filled limit region is ∼0.34 V, and the corresponding trap density is estimated as 7.9 × 10^13^ cm^−3^. This trap density value is four orders of magnitude lower than that observed for polycrystalline films (∼10^17^ cm^−3^) and two orders of magnitude lower than that observed for epitaxially grown perovskite films (∼10^15^ cm^−3^), but higher than that of single crystals (10^9^ to 10^10^ cm^−3^)^22^. Low trap-state density together with high crystallinity of the perovskite films could be responsible for their noteworthy performances of the film as an optical gain medium. Furthermore, it is important to determine the intrinsic carrier mobility of our OPC films. Since the Hall-effect method can investigate the carrier transport that is not significantly influenced by the thickness of active layer or by interfacial properties we observed a high hole mobility of ∼22 cm^2^ V^−1^ s^−1^ and a carrier density of ∼1.0 × 10^12^ cm^−3^ (Supplementary Fig. 13). This relatively high intrinsic mobility of such solution-processed perovskite films opens the possibility to applications such as electrically pumped lasing.

### Film orientation dependent charge transport properties

**Figure 6:**
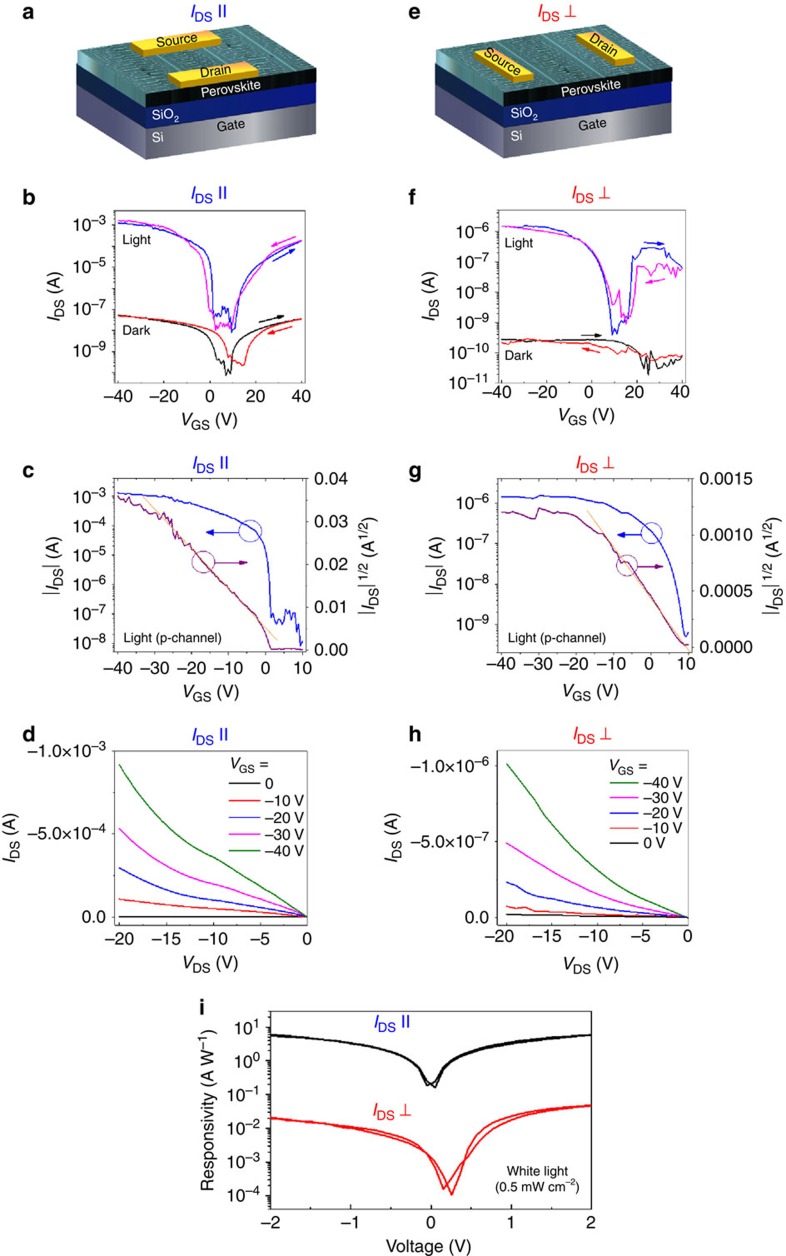
Phototransistor characteristics of the OPC films. (**a**,**e**) Illustrations of transistor structures showing two different geometries of source and drain electrodes deposited in the direction of the backbone and the branch, respectively. (**b,f**) Representative transfer characteristics of aligned OPC films measured along the direction of the main backbone (*I*_DS //_, as illustrated in (**a**)) and in the direction normal to the backbone (*I*_DS_ ⊥, as illustrated in (**e**)), respectively, under both dark and white-light illumination (power density=0.5 mW cm^−2^) conditions at 78 K. Arrows in the graph show the sweep directions. (**c**,**g**) *I*_ds_^1/2^ and *I*_ds_ curves as a function of *V*_gs_ corresponding to **b**,**f** respectively. A linear fit (red line) was used to extract the mobility (*μ*) and threshold voltage (*V*_th_) with the equation of FET devices: *I*_ds_=(*μWC*_0_/2*L*) (*V*_gs_−*V*_th_)^2^. (**d**,**h**) The output characteristics of the devices under illumination. (**i**) Photoresponsivity characteristics measured along the direction of the backbone (black) and the branch (red), respectively.

The electronic properties of perovskites strongly depend on the film morphology, crystallinity and crystal orientation. To investigate how the macroscopic alignment influences charge transport properties, we fabricated field-effect transistor (FET) devices with the aligned OPC films and characterized transport properties in different directions. Top-contact, bottom-gate phototransistor devices with p-Si^++^/SiO_2_/perovskite/Au stacks for two different geometries of source and drain metal contacts deposited along the main backbones (strips) and normal to the backbones are shown in Fig. 6a,e, respectively. We used 2 μm-thick films, grown using the same processing parameters as described above (see the Methods for more details) for all FET experiments.

Figures 6b and f shows the representative transfer data (*I*_ds_−*V*_gs_) of aligned OPC films measured along the direction of the main backbone as illustrated in Fig. 6a (hereafter referred to as *I*_DS //_) and along the direction normal to the backbone as depicted in Fig. 6e (*I*_DS_ ⊥) under both white-light illumination (power density=0.5 mW cm^−2^) and dark conditions. Figure 6c,g shows the plots of *I*_ds_^1/2^ (p-channel) as a function of *V*_gs_ corresponding to Fig. 6b,f, respectively (See Supplementary Fig. 8 for n-channel behavior). A linear fit was used in the saturation regime of the *I*_ds_^1/2^ versus *V*_gs_ curve to extract the mobility (*μ*) and threshold voltage (*V*_th_) with the standard equation of FET devices: *I*_ds_=(*μWC*_0_/2*L*) (*V*_gs_−*V*_th_)^2^, where *W*, *C*_0_ and *L* are the channel width, gate dielectric capacitance per unit area and channel length, respectively. The characteristics of FET devices using aligned films measured in the *I*_DS //_ direction under illumination exhibit typical ambipolar behavior, with a hole mobility (*μ*_h_) of 2.1±0.3 cm^2^ V^−1^ s^−1^ at 78 K (Fig. 6c), close to that measured at 298 K (1.3±0.4 cm^2^ V^−1^ s^−1^, Supplementary Fig. 9b). The on/off ratio (*I*_on/off_) of those devices was ∼10^5^. Note that the mobility we observed is an order of magnitude higher than that of previously reported polycrystalline perovskite films (*μ*_h_=0.18±0.05 cm^2^ V^−1^ s^−1^ at 298 K) fabricated with the same device configuration^8^. The output characteristics, the dependence of *I*_ds_ on *V*_ds_ at different *V*_gs_, of the devices under illumination are shown in Fig. 6d,h. The hole mobility and on/off ratio of the devices measured (at 78 K) in the *I*_DS_⊥direction under illumination were markedly reduced to approximately 8.7 × 10^−3^ cm^2^ V^−1^ s^−1^ and 10^3^, respectively (Fig. 6f,g). Similar FET characteristics (*μ*_h_=6.1 × 10^−3^ cm^2^ V^−1^ s^−1^ and *I*_on/off_ of ∼10^3^,) were observed at 298 K (Supplementary Fig. 9d,e). Under dark conditions at 78 K, FETs measured in the *I*_DS //_ direction showed lower mobilities (*μ*_h_ of ∼8 × 10^−5^ cm^2^ V^−1^ s^−1^) than those measured under illumination, but the *I*_on/off_ of ∼10^3^ is still reasonable, whereas the devices measured with *I*_DS_⊥direction exhibited low device performances with negligible field effect and the *I*_on/off_ of ∼10 (Fig. 6f). The rough transfer curve under dark condition in Fig. 6f is presumably associated with trap populations, grain boundaries, and inferior electron transporting properties of the OPC films (relative to hole transporting properties) through the *I*_DS_⊥direction (See Supplementary Fig. 10 for a more detailed description of the relatively rough transfer curves). The FET characteristics (*I*_DS //_ under dark) worsen when measured at 298 K (*I*_on/off_ of approximately 10, *μ*_h_ of ∼8 × 10^−6^ cm^2^ V^−1^ s^−1^, Supplementary Fig. 9a). We noted that at a lower temperature, hysteresis, which originates from the suppressed screening effect caused by ionic transport, was reduced^44^. Temperature dependent charge transport properties will be addressed in the discussion section below. We also performed the FET measurement with the *I*_DS_ at ∼30° tilted angle with respect to the direction of the backbone (Supplementary Fig. 11). Devices performed better at this angle (*μ*_h_=3.2±0.7 × 10^−2^ cm^2^ V^−1^ s^−1^ at 298 K and 4.3±0.5 × 10^−2^ cm^2^ V^−1^ s^−1^ at 78 K) than in case of *I*_DS_ ⊥, but worse than in case of *I*_DS //_. Results from our measurement of the angle-dependent charge transport properties and the cross-sectional SEM analysis indicate that the anisotropic charge transport properties are dictated by the interconnection areas of the side branches (Fig. 3b). These grain boundaries are responsible for the inferior charge transport properties in the *I*_DS_⊥direction.

Photoresponsivity (*R*) is an indicator of how efficiently an optoelectronic device responds to an optical signal, and is therefore an important figure of merit for evaluating the performance of phototransistors. It is given by *R=*(*I*_light_*−I*_dark_)*/E*_light_, where *I*_light_ and *I*_dark_ are the channel currents under illumination and in dark, respectively, and *E*_light_ is the power of incident illumination. We measured photoresponsivity as a function of *V*_GS_ in our phototransistors, finding a maximum *R* (at 78 K) along the direction of the backbone as 6.1 A W^−1^ (Fig. 6i), which is two orders of magnitude higher than that of the devices measured in the direction of the branches (0.047 A W^−1^). This is consistent with the results of the transfer characteristics measured under dark and illumination conditions.

Overall, our FET measurements lead to the following conclusions. First, the aligned OPC films show considerable anisotropy in field-effect charge mobility depending on the local orientation of the measurement current. Second, the gate-tuning effect is more evident under illuminated compared with dark conditions because the photogenerated charge carriers govern the charge transport process. The suppressed ion migration at lower temperatures gives rise to less hysteresis and higher device performance.

## Discussion

To explain the origin of the branched periodic micropatterns in OPC films, we present a simplified qualitative description of the formation process. We posit that the growth process is controlled by a temperature-dependent solvent evaporation rate, which produces supersaturation of the precursor molecules later consumed by their deposition onto crystal surfaces. The solid-liquid interface moves in the direction of temperature gradient (that is from higher to lower solvent evaporation rate). Consider a simple model of crystallization from a one-component molecular precursor solution. At higher evaporation rates, the concentration of the precursor at the solid–liquid interface, *n*_int_, is higher and hence the crystal growth rate (interface motion velocity) can be defined as


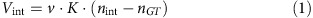


where *v* is the volume of the precursor molecule in the crystal, *K* is the interface reaction rate (kinetic constant), and *n*_GT_ is the Gibbs–Thomson concentration of the precursor molecules. If the pulling velocity on the film is greater than *V*_int_, then more of the solution will be pulled into the higher temperature region with increased solvent evaporation rate. This will increase *n*_int_ and subsequently increase the crystal growth rate. Consequently, the velocity of the interface increases until it enters an area where the concentration of the precursor at the interface decreases; thereafter, the velocity decreases. The process of *V*_int_ variation can be periodic due to the delay between the changes in *n*_int_ and interface motion. We attribute the patterning of our crystalline film to this periodicity. In the presented description we do not consider crystal growth anisotropy, and the orientation of the growth front with respect to the direction of interface propagation. However, the outlined picture helps to understand the origin of the ripples on the crystal film surface as being due to a variation in the growth rate *V*_int_. A more quantitative and detailed description taking into account the kinetic coefficient of anisotropy, the precursor diffusion in the solution and possible liquid dynamics is under investigation.

The recombination rates, carrier density and carrier mobility are inextricably linked together. Analysing different recombination mechanisms is crucial for designing specific strategies to alter each recombination rate by materials engineering. Identifying the dominant recombination process is also important for applications such as solar cells, light emitting diodes and lasers. The dynamics of different carrier recombination processes are described by the universal rate equation:^34^


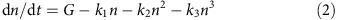


where *n* is the carrier density, *G* is generation rate, and *k*_1_, *k*_2_, and *k*_3_ are monomolecular (due to excitonic or trap-assisted), bimolecular and Auger recombination rate constants, respectively. Regarding the carrier density dependent recombination rates, our results from the TA decay with varying pump fluence clearly revealed that the trap-state mediated monomolecular recombination is dominant at low pump fluences <6 μJ cm^−2^, and the higher order recombinations are dominant within the range of 9–80 μJ cm^−2^. In addition to the TA, a sharp transition from SE to ASE offers a framework for the dominant recombination pathways in the OPC films. Apparently, ASE is effective at a carrier density where *k*_2_ dominates over *k*_1_ and *k*_3_. The relationship between the bimolecular recombination and mobility is simply described by the traditional Langevin theory (*k*_rec_=*eμ*/*ɛ*_o_*ɛ*_r_, where *e*, *μ*, *ɛ*_o_, and *ɛ*_r_ are the elementary charge, carrier mobility, absolute and relative dielectric constant, respectively). The recombination rate of our OPC films derived from the Langevin theory is 1.2 × 10^−6^ cm^3^ s^−1^, which is several orders of higher than the reported values (10^−10^ to 10^−11^ cm^3^ s^−1^)^36^. This deviation with anomalously low recombination rate has been attributed to the splitting of band edge states caused by Rashba–Dresselhaus effect^43^,^45^ and fluctuations in electrostatic potential due to molecular arrangements and octahedral distortion^46^.

With low carrier densities, carrier scattering plays an important role in the charge transport. With the assumption of an ideal electron gas in the solid, the Drude model can describe the carrier mobility (*μ*) with scattering time (*τ*_s_), where *μ*∝*τ*_s_^47^,^48^. The Drude model only considers the interaction of free carriers with its environment, but neglects long-range interactions between electrons or ions^48^. Regarding the scattering mechanisms dominating the free carrier transport in MAPbI_3_ solids, acoustic phonon scattering has been considered as a main factor^49^,^50^. In this case, the mobility is proportional to *T*^−3/2^. This kind of acoustic phonon scattering is well established for non-polar semiconductors such as Si and Ge. However, care must be taken to generalize this to polar perovskites because other scattering sources may participate in the dissipation process. Recent reports revealed that polar longitudinal optical phonon modes could be a dominant source at high temperature in MAPbI_3_ films^51^. Besides phonon scattering, charged defects affect mobility^48^ with an opposite temperature dependence: *T*^+3/2^. In other words, for most solution-processed materials, their mobility could have weaker temperature dependence than anticipated from pure phonon scattering models. Recently, charged point defects in MAPbI_3_ have been rigorously discussed^52^ and their role in free carrier transport cannot be overlooked. In our case, we observed that the mobility estimated from FET measurements at 78 K is an order of magnitude higher than that at RT under dark conditions, meaning that band-like transport, which is limited mainly by phonon scattering, could be dominant despite the existence of trapping. Under illumination, the difference of mobilities between 78 K and RT in fact decreases compared with the dark case, indicating a certain degree of trap filling or existence of other scattering mechanisms when the photo-excited carriers dominate the channel transport. Generally, light illumination increases the PL lifetime and intensity of MAPbI_3_ films, which are attributed to the reduction in trap density. However, our relatively weak light illumination condition (white-light, 50 mW cm^−2^) is quite far from the situation of complete trap filling. We would expect the field effect mobility to be at least one order of magnitude higher if the traps were filled completely, which is quite unlikely for solution-processed materials because the grain boundaries and structural defects always exist regardless of illumination conditions. Therefore, even with perovskite systems of pure crystal orientation, the band-like conduction of charge carriers cannot be realized on the spatial scale of the actual FET devices by comparing the temperature dependence observed in the short-scale mobility of charge carriers.

In conclusion, we have demonstrated the controllability of thin film directional crystal growth in a scalable manner and the preservation of high crystallinity and orientational purity during crystal growth through a novel thermal gradient-assisted directional crystallization method. The method we designed results in OPC films that are clearly distinguishable from previously reported thin film crystallization methods of organolead halide perovskites. Our main findings on OPC films include unique crystal orientation, low trap-state density giving rise to strong ASE, and the significant anisotropy in charge transport. The demonstration of perovskite OPC film based phototransistors and optical gain media provides an essential guideline for materials optimization through crystallization processing and the future developments of novel optoelectronic devices.

## Methods

### Reagents

Methylamine (33% in absolute ethanol) and formic acid (98% assay) were purchased from Aldrich. Methylammonium iodide (MAI) was purchased from Dyesol. Lead iodide (99.999 trace metal basis) was purchased from Alfa Aesa. All chemicals were used without further purification.

### Synthesis of MAFa and directional crystal growth

The synthesis of MAFa has previously been described^24^,^53^. Briefly, 3 ml of formic acid diluted with absolute methanol (12.5 ml) was slowly added to a round flask containing 12.5 mL of methylamine (33% in ethanol) and 5 ml of absolute ethanol at −16 °C. The reaction mixture was stirred for 2 h, and then the residual solvent was removed by a rotary evaporator at 30 °C. A clear, viscous liquid was obtained. A 25 wt% solution of lead iodide and MAI (1:1 molar ratio) in MAFa was prepared and stirred for 1 h at room temperature. The aligned microarray films were prepared by blade coating of the solution at room temperature on ITO or p-Si^++^/SiO_2_ substrates (Crystal orientation is not related to the direction of blade coating). After blade coating at room temperature, the edge of the substrate (∼5 mm) was attached on top of the annealed metal bar by Kapton tape, as shown in Fig. 1a. The entire substrate was covered with a crystallization dish to prevent airflow, which can disturb the uniform solidification process. Typically, 2 h of solidification time at 85 °C was required to grow 1.5-cm-long microarrays. After directional solidification, the films were further annealed on a hot plate for 2 h. It is worth mentioning that a simple drop casting method can also make nicely oriented films, but controlling their thicknesses is difficult with such a method. Note that the plasma cleaning of the substrate is an essential step for making nicely oriented films because wetting (surface energy matching) between the precursor solution and the substrate is one of the critical factors for homogeneous directional growth.

### Characterization

The crystal structure of perovskite films was analysed using an X-ray diffractometer, Bruker AXS D8, with CuKα radiation. Absorption and photoluminescence were measured by a Varian Cary 5000 spectrometer and an Edinburgh FLS920, respectively. Keithley 2400 was used to perform *I*–*V* characteristics. TA decays were measured with a femto-nanosecond pump-probe setup. The detailed experimental setup has been described elsewhere^54^,^55^. To estimate the lifetime (*t*) at higher excitation intensities, the data were fitted with a bi-exponential decay function (*y*=*y*_0_+*A*_1_e^−(*x*−*x*0)/*t*1^+*A*_2_e^−(*x*−*x*0)/*t*2^), while at lower excitation intensities, the data were fitted with a single-exponential decay (*y*=*y*_0_+*A*_1_e^−(*x*−*x*0)/*t*1^). The obtained time components are provided in Supplementary Table 1.

All ASE pumping experiments were conducted at room temperature. The one-photon pumping (650 nm) experiments were performed using a femtosecond laser system operated at a wavelength 800 nm with 35 fs pulses and a repetition rate of 1 kHz. A Quanta 200 FEG instrument was used to obtain SEM images. A TitanG2 80–300 instrument, FEI Co., Super Twin, x-FEG, operating at 300 kV was used for TEM analysis. The perovskite film dispersions in toluene were sonicated for five seconds, then deposited onto copper grids. Samples were dried in air for 1 h before measurements were taken. The phototransistor and photodetector were characterized using a Keithley 4200 semiconductor parametric analyser and a Signaton Micromanipulator S-1160 probe station. A white-light-emitting diode (0.5 mW cm^−2^) attached to the microscope of the probe station was applied as the light source. The dielectric constant measurement was carried out using a Keithley 4200 semiconductor parameter analyser at room temperature in dark condition. Direct current voltage was applied to the devices (ITO/Perovskite/Au) from −1 to +1 V in 0.02 steps. The applied frequency range was from 1 kHz to 1 MHz with 25 mV of alternating currentvoltage perturbation. All measurements were carried out under vacuum. Hall-effect measurements were performed using a Lakeshore model 665 system at room temperature on a 4-probe sample holder placed between the plates of an electromagnet. The samples were measured under a magnetic field varied in the 10–15 kG range.

### Data availability

The data that support the findings of this study are available from the corresponding author on reasonable request.

## Additional information

**How to cite this article:** Cho, N. *et al*. Pure crystal orientation and anisotropic charge transport in large-area hybrid perovskite films. *Nat. Commun.* **7,** 13407 doi: 10.1038/ncomms13407 (2016).

## Supplementary Material

Supplementary Information Supplementary Figures 1-13, Supplementary Table 1 and Supplementary Notes 1 & 2.
