# Supplementary material for: Pure crystal orientation and anisotropic charge transport in large-area hybrid perovskite films
Source: Nat Commun. 2016 Nov 10;7:13407. doi: 10.1038/ncomms13407 (PMC5109592; doi:10.1038/ncomms13407)
Supplement: Supplementary Information — Supplementary Figures 1-13, Supplementary Table 1 and Supplementary Notes 1 & 2. [file ncomms13407-s1.pdf]

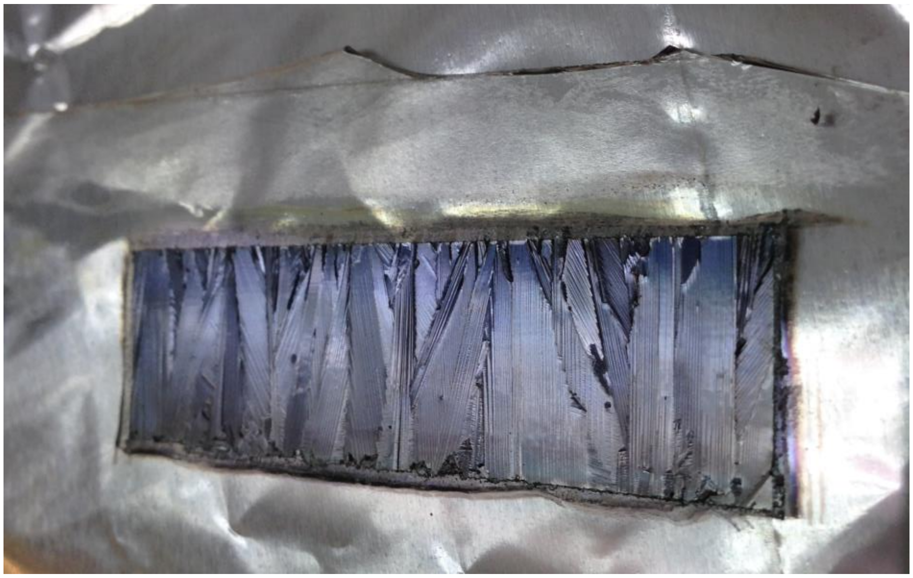

Supplementary Figure 1. A photographic image of directionally grown perovskite films on a glass substrate (size: 1.5 × 4.5 cm).

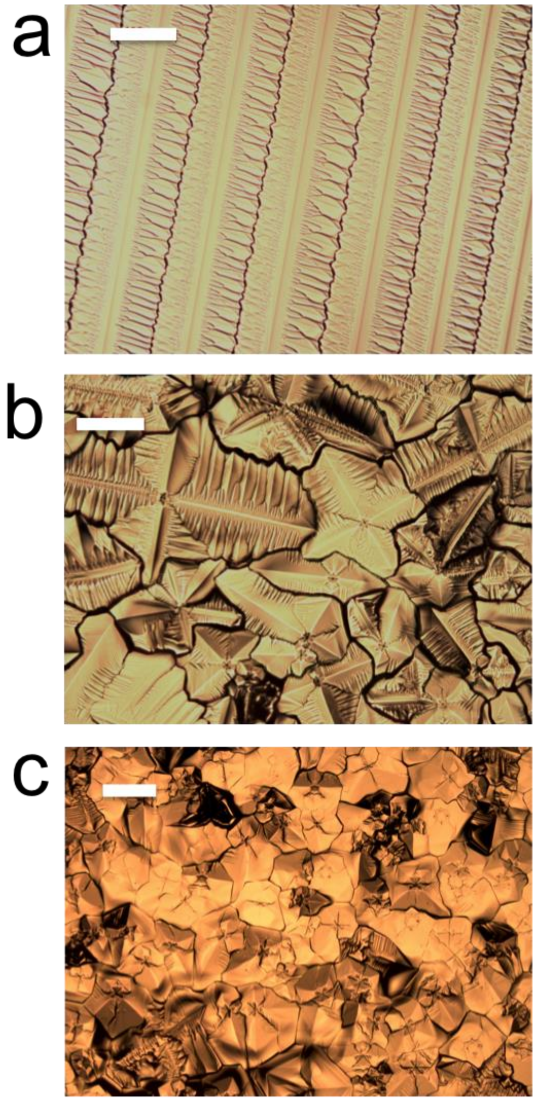

Supplementary Figure 2. Optical microscope images of $MAPbI_3$ films formed with different concentrations: (a) 50 wt.%, (b) 125 wt.%, and (c) 150 wt.%. Scale bars for (a), (b), and (c) are 100, 200, and 300 μm, respectively.

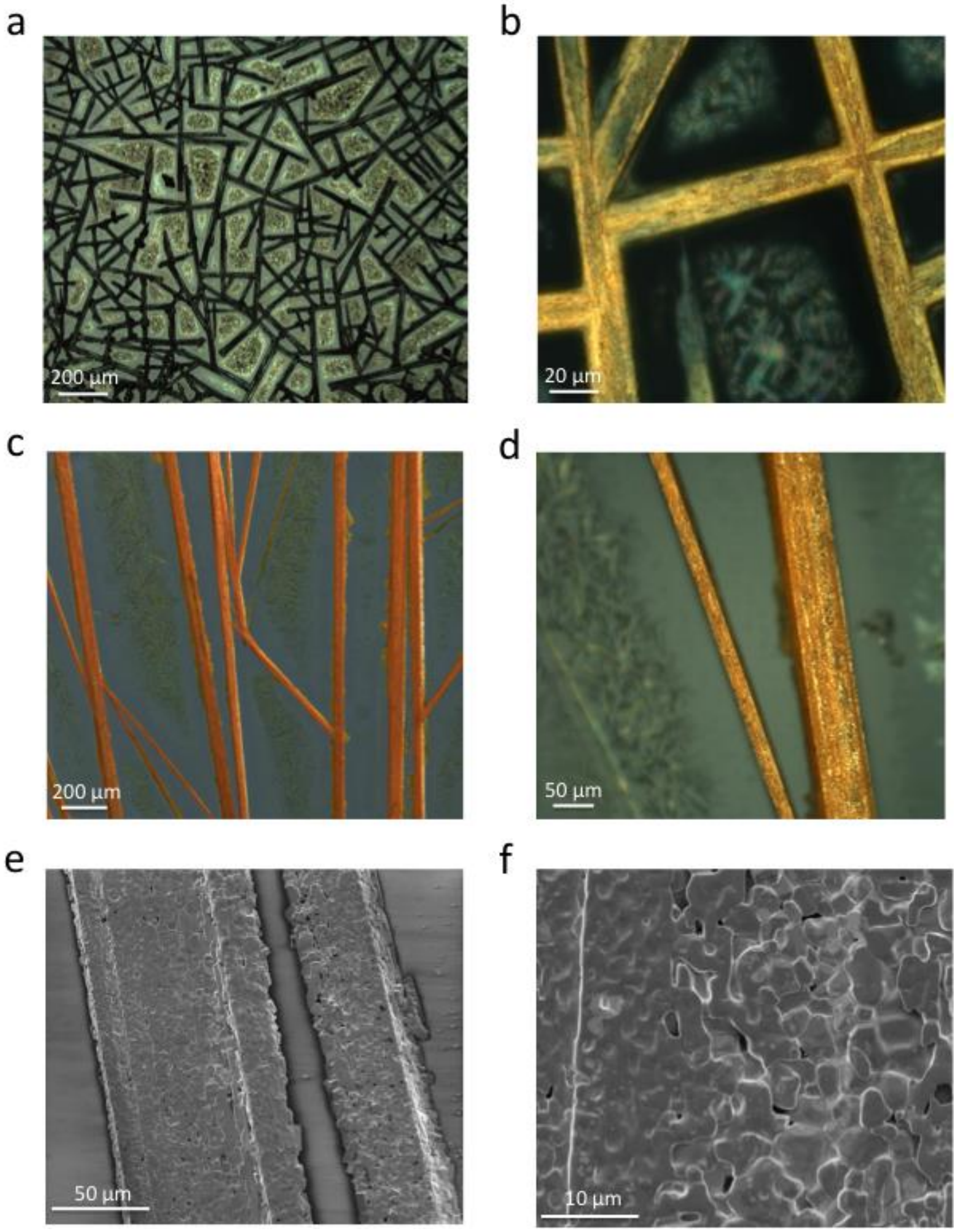

Supplementary Figure 3. (a) Optical microscope image of randomly grown $MAPbBr_3$ wires with homogeneous annealing and (b) their magnified image. (c) Optical microscope image of directionally grown $MAPbBr_3$ wires using the thermal gradient method and (d) their magnified image. (e) SEM image of directionally grown wires showing their polycrystalline nature and (f) their SEM image in higher resolution.

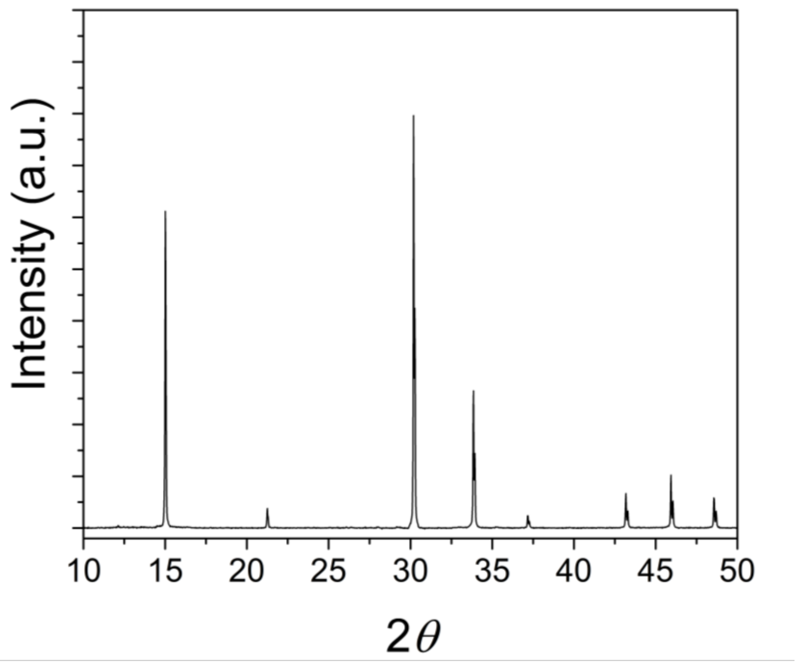

Supplementary Figure 4. X-ray diffraction of the directionally grown $MAPbBr_3$ wires using the thermal gradient method.

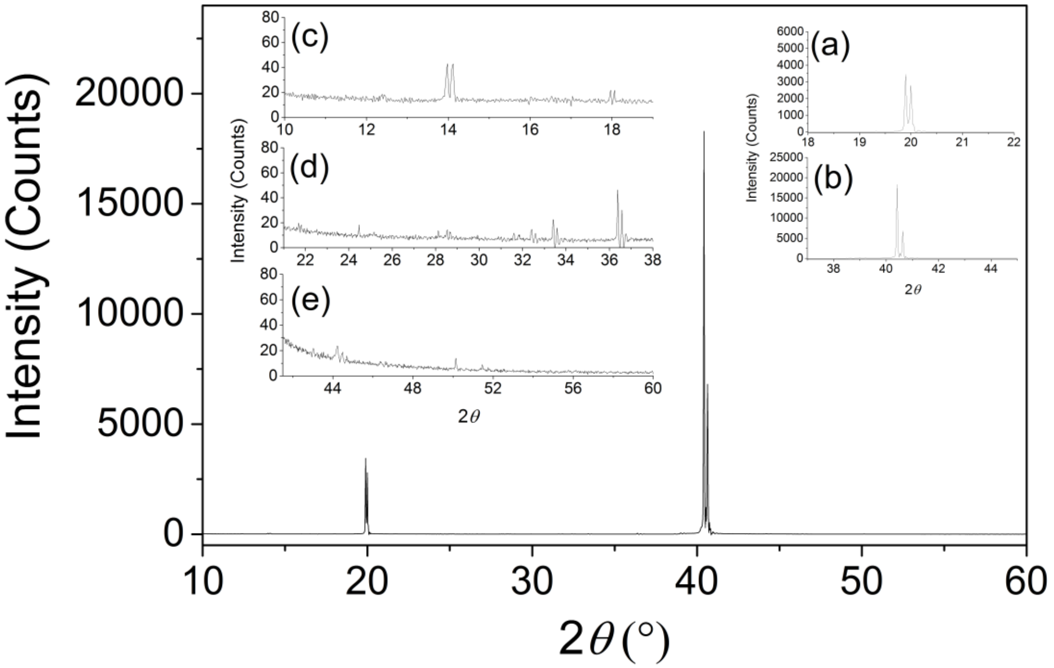

Supplementary Figure 5. X-ray diffraction analysis of the perovskite OPC films showing detailed XRD peaks. XRD analysis in the baseline level shows small peaks with negligible XRD intensities (inset Fig. c, d, and e) compared to strong intensities from the (112) and (200) peaks (inset Fig. a and b).

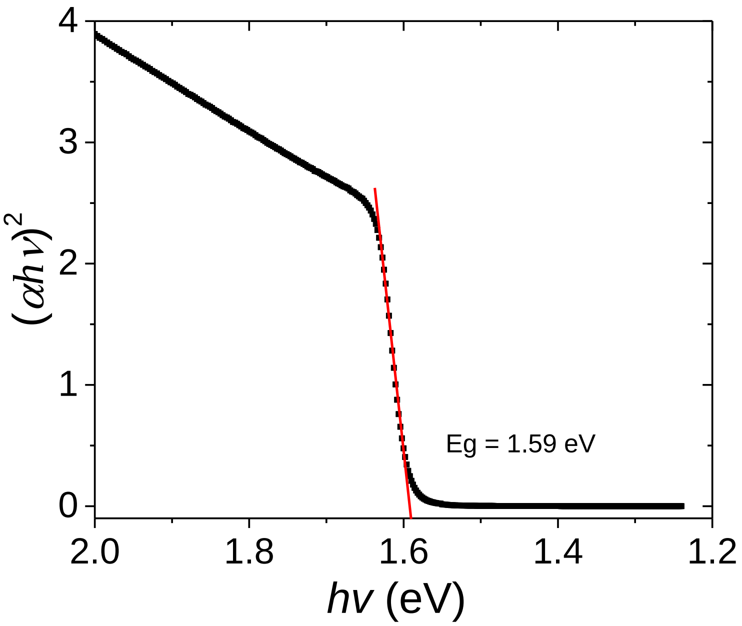

Supplementary Figure 6. Tauc plot from the absorption spectrum of an aligned microarray film showing the extrapolated optical band gaps.

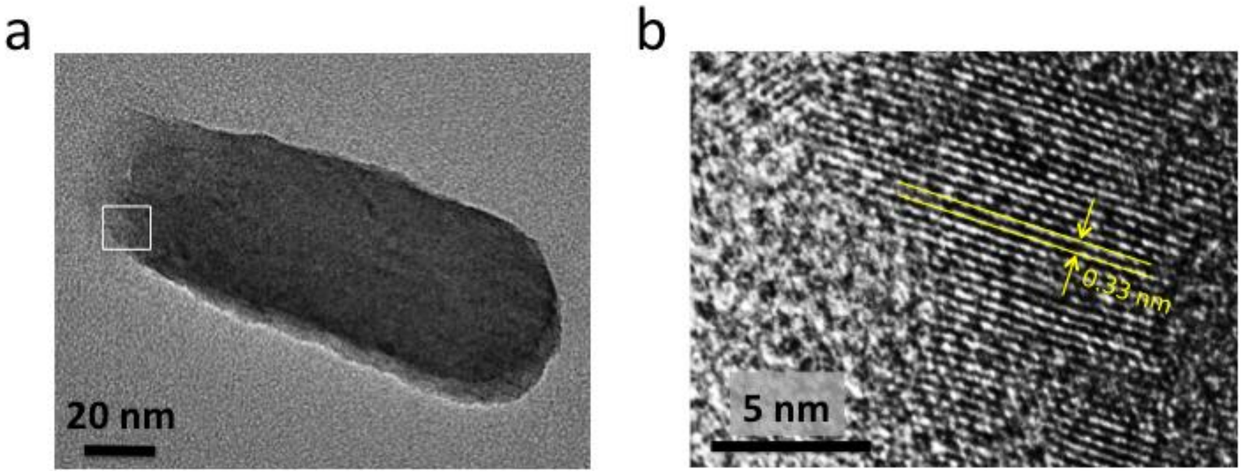

Supplementary Figure 7. (a) TEM image of a small parallel branch separated from the OPC films. (b) High-resolution TEM image of the highlighted area in (a) (white square). The crystalline structure with a lattice spacing of 0.33 nm was observed.

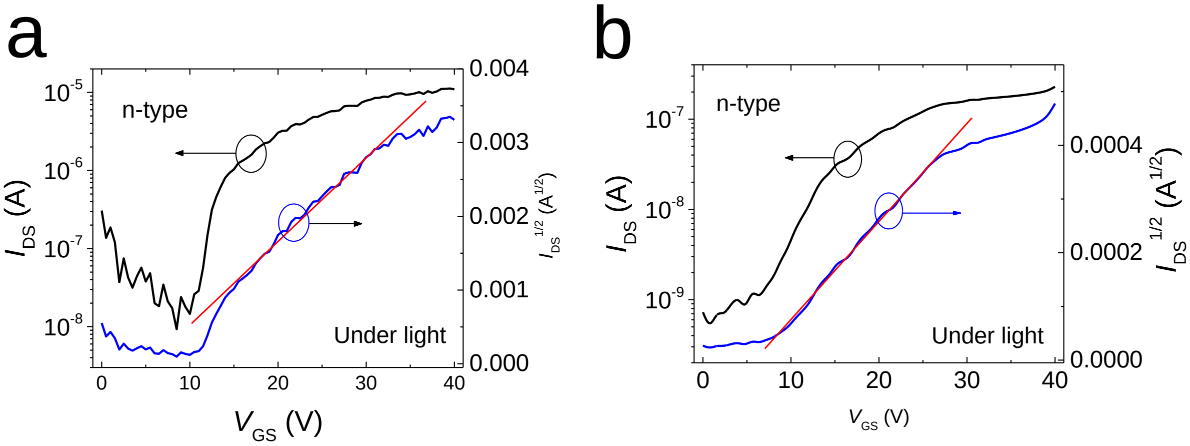

Supplementary Figure 8. N-channel V_{GS}-$I_{DS}^{1/2}$ characteristics of the aligned OPC films measured at 78 K under illumination measured with (a) $I_{DS\,//}$ and (b) $I_{DS\,\perp}$ directions. A linear fit (red line) was used to extract the mobility (μ) and threshold voltage (V_{th}) with the equation of FET devices: $I_{ds} = (\mu W C_0/2L)\,(V_{gs} - V_{th})^2$.

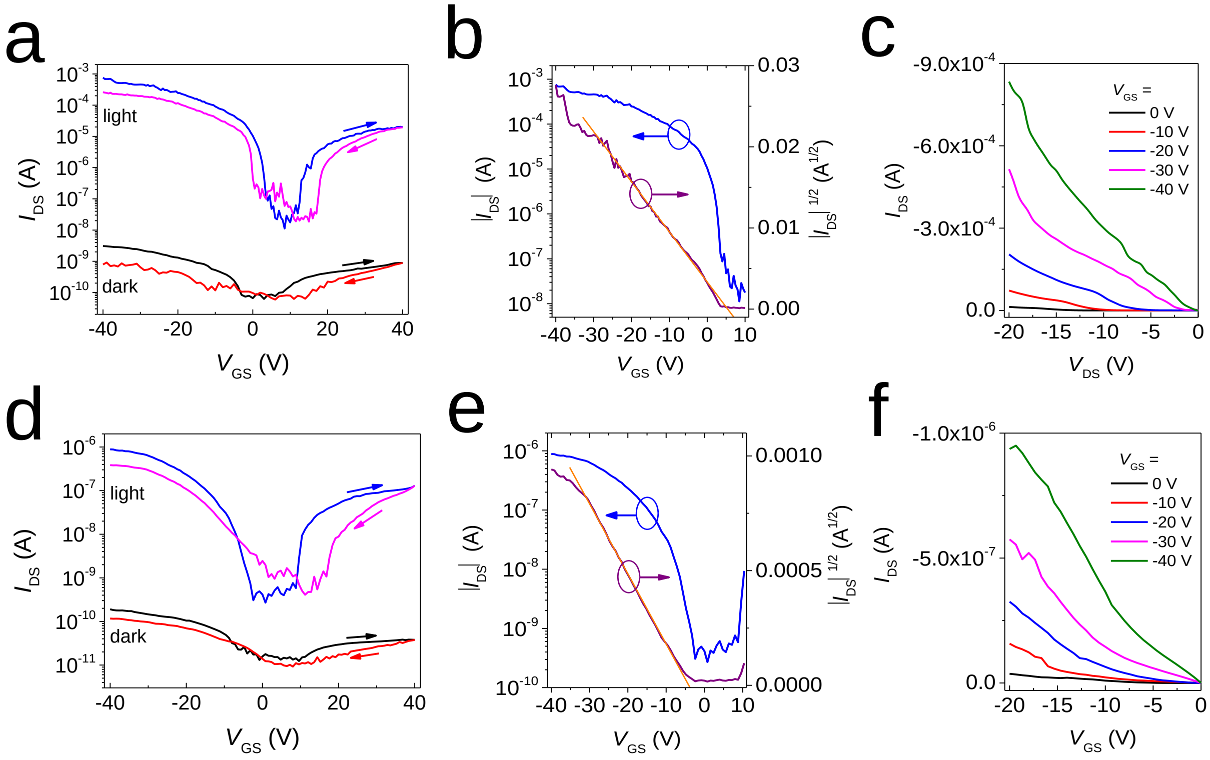

Supplementary Figure 9. (a, d) Representative transfer characteristics of aligned OPC films measured along the direction of the backbone ($I_{DS\,//}$) and normal to the backbone ($I_{DS\,\perp}$), respectively, under both dark and white-light illumination (power density = 0.5 mW cm^{-2}) conditions at 298K. Arrows in the graph show the sweep directions. (b, e) $I_{ds}^{1/2}$ and I_{ds} curves as a function of V_{gs} corresponding to Figure S9a and S9d, respectively. A linear fit (red line) was used to extract the mobility (μ) and threshold voltage (V_{th}) with the equation of FET devices: $I_{ds} = (\mu W C_0/2L)\,(V_{gs} - V_{th})^2$. (c, f) The output characteristics of the devices under illumination.

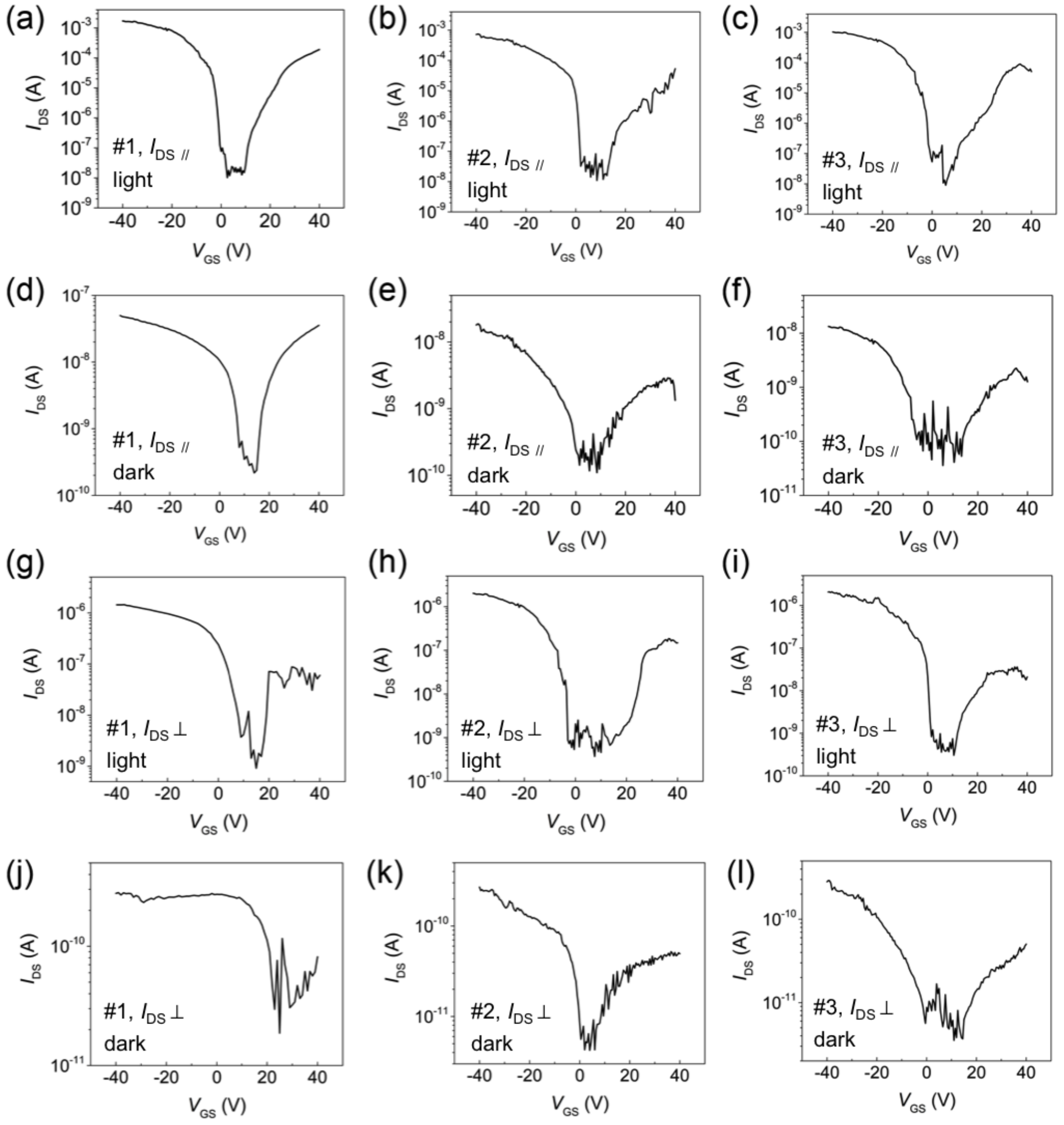

Supplementary Figure 10. Typical transfer curves of aligned OPC films measured from three different devices showing different levels of noise at 78K. (a, b, c) $I_{DS\ //}$ under illumination condition. (d, e, f) $I_{DS\ //}$ under dark condition. (g, h, i) $I_{DS} \perp$ under illumination condition. (j, k, l) $I_{DS} \perp$ under dark condition.

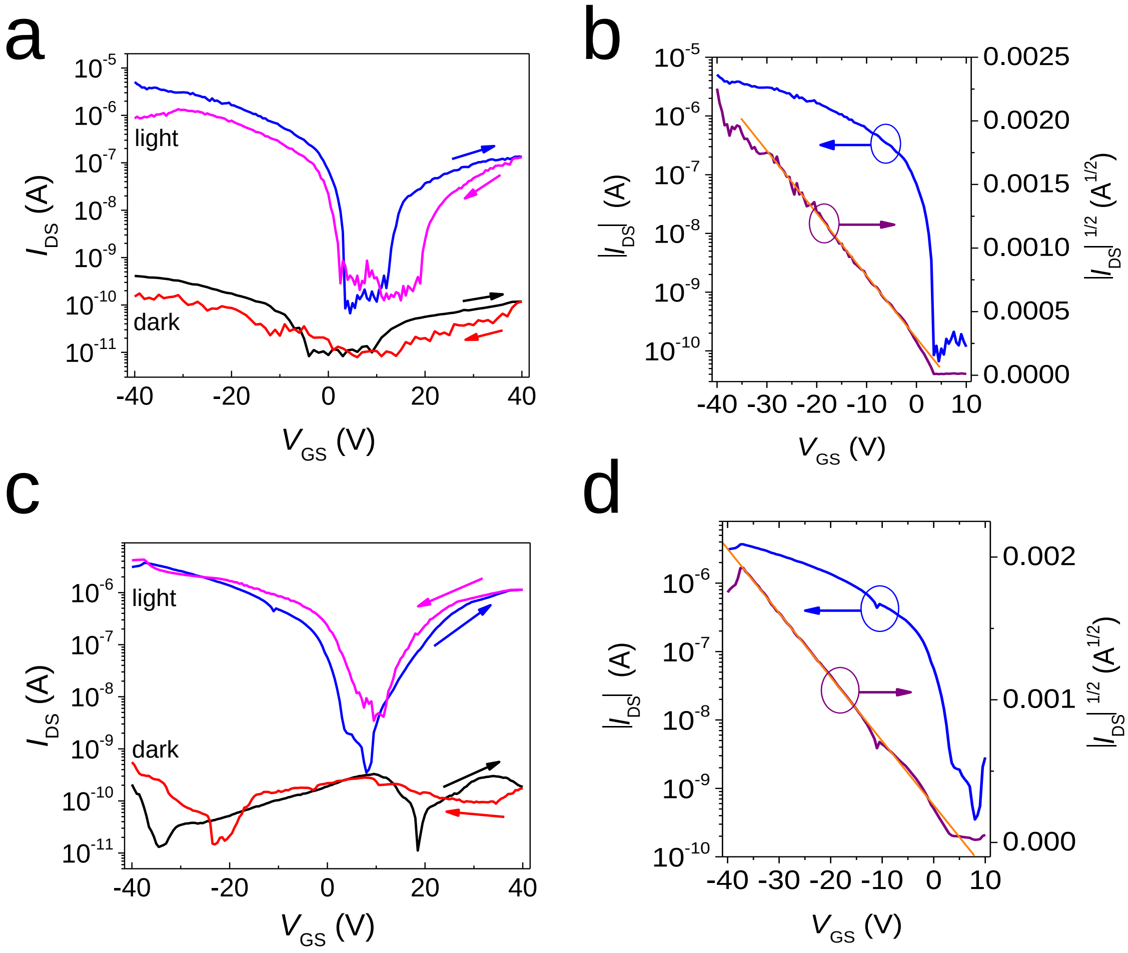

Supplementary Figure 11. (a, c) Representative transfer characteristics of aligned OPC films measured with a 30° angle between the backbone and I_{DS} flow at 78 K and 298 K, respectively. Arrows in the graph show sweep direction. (b, d) $I_{ds}^{1/2}$ and I_{ds} curves as a function of V_{gs} corresponding to (a) and (c), respectively. A linear fit (red line) was used to extract the mobility (μ) and threshold voltage (V_{th}) with the equation of FET devices: $I_{ds} = (\mu W C_0/2L)\,(V_{gs} - V_{th})^2$.

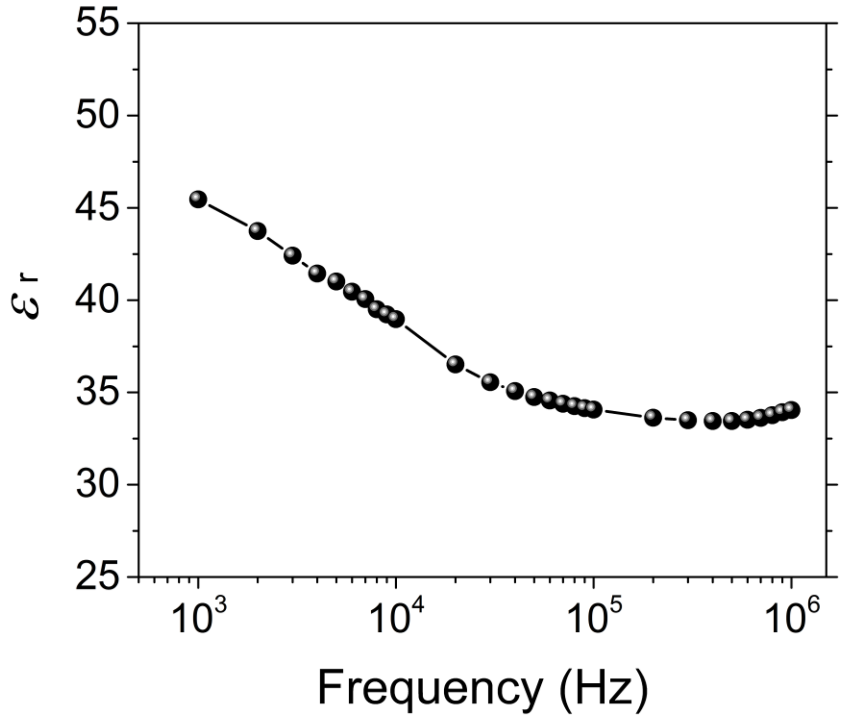

Supplementary Figure 12. Relative dielectric constant of aligned OPC films as a function of frequency measured under dark at room temperature.

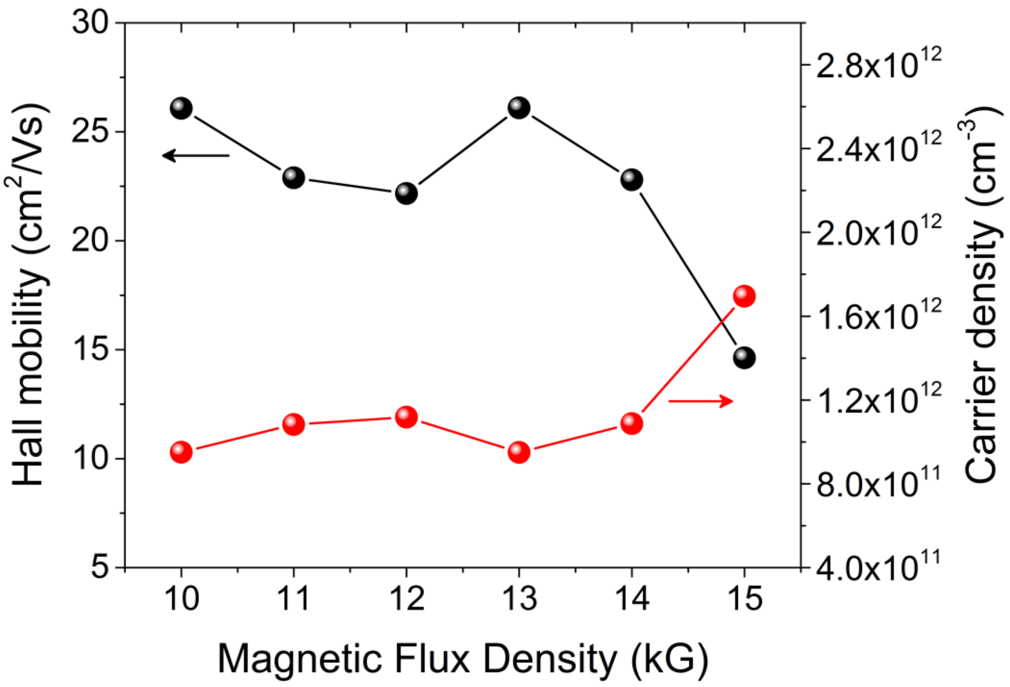

Supplementary Figure 13. The mobility and carrier density characterized by Hall-effect measurement under the magnetic field varied in the 10 - 15 kG range.

Supplementary Table 1. Time constants as estimated from the fit of the kinetic traces.

Pump fluence ($\mu J\ cm^{-2}$)	Amp (%)	t_1 (ns)	Amp (%)	t_2 (ns)
3	-	-	100	430 ±32
5	-	-	100	426 ±38
9	34	26 ±4	66	230 ±16
16	41	18 ±2	59	180 ±5
40	60	9 ±1	40	120 ±3.8
80	68	4 ±0.1	32	79 ±2.5

Supplementary Note 1: As shown in Supplementary Fig. 3 and Supplementary Fig. 4, the morphology and crystallographic property of $MAPbBr_3$ are quit different compared to $MAPbI_3$, and the $MAPbBr_3$ has normal crystallinity as observed from polycrystalline films. The different film morphology and crystallinity can be attributed to the starting compound or crystallographic nature. We think different solubility of $PbBr_2$ compared to PbI_2 in ionic liquid could be one of the important parameters for different morphological and crystallographic properties. The maximum solubility of $PbBr_2$ and PbI_2 in MAFa is ~ 0.7 g ml^{-1} and 1.0 g ml^{-1} at 80 °C, respectively. This difference between two starting materials might cause significant differences in materials properties. To generate a pure crystal orientation we need careful control of the nucleation of perovskite from PbI_2 (or $PbBr_2$) and the efficient intercalation of MAI molecules into the growing PbI_2 framework. Due to the ionic character of MAFa and its strong solvation between COO^- and Pb^{2+} and $CH_3NH_3^+$ and I^-, it dissolves PbI_2 better than other common solvents, which is

responsible for the slow crystallization of lead halide during our directional crystallization process. In general, $MAPbBr_3$ crystals grow much easier and faster than $MAPbI_3$ crystals. Therefore, in the case of directional growth of $MAPbBr_3$, different solubility or faster nucleation/growth rate interrupts the desired reaction rate required for slow and balanced crystallization process.

Supplementary Note 2: In Supplementary Fig. 10, the noises are mainly observed in n-type transfer curves for all devices. This result indicates that the OPC films have inferior electron transporting properties. In addition, the devices measured with $I_{DS} \perp$ direction under dark condition (Supplementary Fig. 10j, k, l) show very rough transfer curves. In this case, we were not able to estimate the carrier mobility. The grain boundaries, trap populations, and insignificant electron transport properties are probably responsible for this.
